# The involvement of human CD33 in the pathogenesis of Alzheimer’s disease mouse model

**DOI:** 10.1002/alz.086747

**Published:** 2025-01-03

**Authors:** Tzu‐Ching Su, Wei‐Chien Hung, Irene Han‐Juo Cheng

**Affiliations:** ^1^ Institute of Brain Sciene, National Yang Ming Chiao Tung University, Taipei Taiwan

## Abstract

**Background:**

Genome‐wide association studies demonstrated that immune suppressive receptor CD33 variants are associated with high susceptibility to developing Alzheimer’s disease (AD). Human CD33 (hCD33) regulates microglial immune response and clearance ability. However, the differential regulation of phagocytosis by human and mouse CD33 imposes constraints on utilizing the mouse model for investigating the role of CD33 in AD. Therefore, we investigate the pathogenesis progression of the AD mouse model with hCD33 knockin.

**Method:**

We generated conditional hCD33 knock‐in mice on microglia in the J20 AD mouse model. At 6 months, Morris water maze and Barnes maze were applied to examine their spatial learning and memory. The ELISA and immunohistochemistry were used to examine the accumulation of soluble and insoluble Aβ, microglial activation, *in‐vivo* Aβ uptake, and synaptic dysregulation.

**Result:**

In the presence of hCD33, Aβ accumulation was decreased in the AD mouse. Conversely, a more diffuse and less condensed plaque structure was observed. In hCD33 knock‐in mice, microglia failed to form a compact barrier covering plaque deposits. Under hCD33 expression, AD mice had more neurotoxic dystrophic neurite around plaques, suggesting a higher plaque‐associated neurotoxicity. However, spatial learning and memory retention had no significant differences between the control AD mice and hCD33 knock‐in AD mice in the Morris water maze.

**Conclusion:**

Our findings suggested that the presence of the AD risk gene human CD33 incurred neurotoxicity and altered the structure of Aβ deposition by manipulating microglial barrier formation against AD stress.

